# HOX gene regulation in glioma stem cells, mediators of resistance in breast cancer, and paradoxical therapeutic interventions

**DOI:** 10.1002/1878-0261.13059

**Published:** 2021-08-03

**Authors:** Ruzhica Bogeska

Therapy resistance remains a major challenge during the treatment of patients with advanced cancer. Due to the complex nature of the cellular and molecular mechanisms underlying therapy resistance, research efforts to tackle this challenge have focused on diverse aspects: from cancer stem cell (CSC) biology, therapy resistance‐specific signatures to the development of new combinatorial therapeutic approaches. Among other articles, the August issue of *Molecular Oncology* includes several studies that directly or indirectly explore therapy resistance in patients with advanced cancer.

In some cancers, therapeutic resistance has been linked to the presence of CSCs. In glioma, for example, glioma stem cells (GSCs) represent a tumor cell subpopulation underlying tumor relapse. Understanding the molecular mechanisms behind GSC gene expression may give new insights on tumorigenesis and tumor relapse. In their recent study, Le Boiteux *et al*. [1] analyzed six patient‐derived GSC lines, as well as 70 primary adult diffuse glioma samples to map the mechanisms leading to deregulation of the HOX gene clusters, which have been previously implicated in the oncogenic potential of GSCs. The authors identified widespread reactivation of HOX genes in adult IDH wild‐type (IDHwt) diffuse glioma samples and GSCs. HOX gene deregulation was associated with self‐ and cross‐regulatory interactions between HOX transcription factors, but not with genomic rearrangements. Integrative analysis of high‐throughput DNA methylation analysis, ChIPseq, and RNAseq data demonstrated that the absence of hypermethylation on canonical and alternative bivalent CpG islands in promoters, as well as loss of the H3K27me3 epigenetic mark, underlies aberrant gene expression of HOX clusters in IDHwt gliomas. Besides providing a comprehensive epigenetic analysis of HOX gene regulation in glioma, the study by Le Boiteux *et al*. highlights the complexity of HOX gene expression via alternative promoters in cancer.

In a different setting, Mohammad Sultan and colleagues employed an *in vivo* genome‐wide shRNA screen to identify potential mediators or biomarkers of paclitaxel resistance in patients with advanced breast cancer [2]. The transcriptional repressor BCL6 was identified among the top paclitaxel resistance hits, and BCL6 overexpression was detected in tumors of chemotherapy‐resistant patients. To further test the role of BCL6 in paclitaxel resistance, the authors performed *BCL6* knockdown and BCL6 inhibitor experiments both *in vitro* and *in vivo*, in a mouse model of triple‐negative breast cancer. BCL6 targeting in preclinical models enhanced the efficacy of paclitaxel treatment. Thus, BCL6 may serve as a biomarker for drug resistance and may potentially be targeted for the treatment of patients with breast cancer that have developed paclitaxel resistance.

In addition to chemotherapy, targeted therapies designed to block mitogenic signaling pathways are often used for the treatment of patients with cancer. However, drug resistance to these therapeutic agents represents a major challenge for the treatment of advanced cancer. In their perspective, Matheus Henrique Dias and Rene Bernards explore the prospects of a ‘paradoxical intervention’ strategy for the treatment of therapy‐resistant cancers [3]. Such a strategy would involve overactivation, rather than inhibition, of mitotic signaling in cancer cells. In their view, such an approach would result in an overload of the stress response pathways and disruption of cancer cell homeostasis and viability. The authors review related data from several independent experimental models, discuss future perspectives, and explore the potential drawbacks of such a paradoxical intervention strategy for the treatment of patients with cancer.

## References

[1] Le Boiteux E , Court F , Guichet PO , Vaurs‐Barrière C , Vaillant I , Chautard E , Verrelle P , Costa BM , Karayan‐Tapon L , Fogli A *et al*. (2021) Widespread overexpression from the four DNA hypermethylated HOX clusters in aggressive (IDHwt) glioma is associated with H3K27me3 depletion and alternative promoter usage. Mol Oncol 15, 1995–2012. 10.1002/1878-0261.12944 PMC8334257 33720519

[2] Sultan M , Nearing JT , Brown JM , Huynh TT , Cruickshank BM , Lamoureaux E , Vidovic D , Dahn ML , Fernando W , Coyle KM *et al*. (2021) An *in vivo* genome‐wide shRNA screen identifies BCL6 as a targetable biomarker of paclitaxel resistance in breast cancer. Mol Oncol 15, 2046–2064. 10.1002/1878-0261.12964 PMC8333778 33932086

[3] Dias MH & Bernards R (2021) Playing cancer at its own game: activating mitogenic signaling as a paradoxical intervention. Mol Oncol 15, 1975–1985. 10.1002/1878-0261.12979 PMC8333773 33955157

